# The Gut Virome in Gastrointestinal Cancers: Mechanisms, Biomarkers, and Translational Opportunities

**DOI:** 10.3390/v18101131

**Published:** 2026-10-12

**Authors:** Michele Ghidini, Giuseppe Sberna, Luigi Pio Guerrera, Fabrizio Maggi, Federica Novazzi

**Affiliations:** 1 Department of Medicine and Technological Innovation, University of Insubria, 21100 Varese, Italy; michele.ghidini@uninsubria.it; 2 Oncology Unit, Azienda Socio Sanitaria Territoriale Sette Laghi, 21100 Varese, Italy; luigipio.guerrera@asst-settelaghi.it; 3 Laboratory of Virology, National Institute for Infectious Diseases “Lazzaro Spallanzani” IRCCS, 00118 Rome, Italyfabrizio.maggi@inmi.it (F.M.); 4 Laboratory of Medical Microbiology and Virology, University Hospital of Varese, Azienda Socio Sanitaria Territoriale Sette Laghi, 21100 Varese, Italy

**Keywords:** gut virome, gastrointestinal cancer, colorectal cancer, bacteriophages, metagenomics, viral biomarkers, phage–bacteria interactions, oncogenic viruses, anelloviruses, translational oncology

## Abstract

The gastrointestinal tract contains a dense multi-kingdom ecosystem in which bacteria, fungi, archaea, viruses, the mucosa, and the immune system interact. Although cancer microbiome research has focused mainly on bacteria, the gut virome dominated by bacteriophages may influence gastrointestinal carcinogenesis by reshaping bacterial communities, transferring functional genes, modulating inflammation, and altering treatment response. The strongest evidence comes from colorectal cancer, where multiple cohorts show altered phage diversity, temperate-phage enrichment, disrupted phage–bacteria networks, and reproducible stool-based diagnostic signals. Emerging studies in pancreatic cancer, esophageal adenocarcinoma, hepatocellular carcinoma, and precursor lesions suggest that virome disruption extends beyond colorectal cancer, although evidence remains uneven. Classic oncogenic viruses, including Epstein–Barr virus and hepatitis viruses, provide complementary examples of direct viral carcinogenesis in digestive organs. Anelloviruses are widespread in healthy individuals and are not established causes of cancer, but their abundance and composition may reflect immune status, inflammation, tissue injury, and altered host viral ecology. Virome-related interventions remain clinically immature: fecal microbiota transplantation has reached small phase I/II studies in gastrointestinal cancers, whereas phage, fecal-filtrate, and fecal virome approaches remain preclinical or non-oncology; direct oncolytic viruses have reached late-phase testing. Major barriers include viral dark matter, specimen and assay heterogeneity, disease-related confounding, limited external validation, and weak causal evidence. Standardized, mechanistically anchored, multi-kingdom studies are needed to establish clinical utility.

## 1. Introduction

The gastrointestinal tract contains a dense multi-kingdom ecosystem in which bacteria, fungi, archaea, and viruses interact with the mucosa and immune system. In cancer research, bacterial dysbiosis has drawn most attention, but this focus obscures a simpler fact: the viral fraction is not small noise layered on top of the bacteriome. It is a major ecological force, dominated in stool by bacteriophages that regulate bacterial fitness, horizontal gene transfer, and community turnover [1,2]. Longitudinal and comparative studies also show that gut viromes are highly individualized and can be stable within individuals, which is important when interpreting disease-associated shifts [3,4].

The gut virome matters for gastrointestinal cancers through two linked routes. The first is mechanistic. Phages can shift bacterial niches, promote the carriage of virulence-associated functions, and alter inflammatory tone, while eukaryotic viruses can directly engage host cell-cycle, survival, and immune pathways [5,6,7]. The second is clinical. Viral signatures in stool, tissue, and blood are increasingly detectable by metagenomics, virus-like-particle enrichment, transcriptomics, and serology, and several studies report useful diagnostic, prognostic, or risk-stratification performance [8,9,10,11].

The field is strongest in colorectal cancer, where repeated cohorts support a phage-centered model of dysbiosis. Outside colorectal cancer, the literature is smaller and more heterogeneous, but it points in the same broad direction: the viral component of dysbiosis is linked to tumor-promoting bacterial ecologies, mucosal inflammation, and host response. Classic oncogenic viruses remain part of this story, not as a rival literature but as proof that viral carcinogenesis in digestive tissues can be direct, indirect, or both [7,12,13].

This review uses the term intestinal virome for the collection of viruses in the gastrointestinal tract, mainly bacteriophages. It separates four mechanistic routes: phage-mediated bacterial dysbiosis; virome-associated inflammation and immune remodeling; lysogeny, horizontal gene transfer, and virulence cargo; and direct viral carcinogenesis. It then examines anelloviruses, cancer-specific evidence, biomarker applications, analytical bottlenecks, and the current therapeutic landscape. The central distinction throughout is between association and causation, and between stool, tissue, and serum measurements, which represent different biological layers.

## 2. The Intestinal Virome as an Ecological Layer

The intestinal virome is not a single uniform compartment. Its composition depends on age, diet, geography, host health, bacterial hosts, sampling location, and the method used to recover viral sequences [1,2]. Stool viromes are dominated by bacteriophages, while eukaryotic viruses are generally less abundant but may have direct effects on host tissue. A large fraction of viral sequences remains unclassified, creating a substantial reservoir of viral dark matter.

Phages can follow lytic or lysogenic cycles. Lytic replication can change bacterial abundance and release bacterial products, whereas lysogeny can alter bacterial phenotype and facilitate the persistence or transfer of accessory genes. These processes make the relevant biological unit a virus–bacterium–host network rather than an isolated viral taxon. A disease-associated phage may be important because it changes the abundance of a bacterial host, co-occurs with a pathogenic community, carries a functional element, or contributes to host immune sensing.

Virome compartments and specimen interpretation: The same viral sequence can have different meanings in different biological compartments; accordingly, this review distinguishes four interpretive layers, as summarized in Table 1.

Stool-derived phage associations, mucosa-associated signals, tumor-resident eukaryotic viruses, and serum antibody signatures should therefore not be pooled into a single undifferentiated virome measure. They are complementary biological layers, but each requires its own sampling controls and causal interpretation. Failure to detect a viral sequence should not be interpreted as evidence of biological absence: recovery depends on specimen handling, viral enrichment, nuclease treatment, nucleic-acid extraction, sequencing depth, library preparation, assembly, read-mapping thresholds, and reference databases [14,15,16]. Mucosa-associated sampling also captures a different biogeographic compartment from stool [17].

### 2.1. Evidence Tiers for Causal Interpretation

The mechanisms discussed below should not be assigned the same causal status. Three evidence tiers help distinguish functional support from association and established viral carcinogenesis Table 2.

This hierarchy is used throughout the review. Phage-associated dysbiosis and immune remodeling are presented as biologically plausible or experimentally supported mechanisms, but most human virome signatures remain associative. EBV, HBV, and HCV provide a separate reference point: their links to digestive malignancy are etiologically established, even though the balance between direct viral effects and inflammation-mediated injury differs among viruses and tissues.

### 2.2. The Virome Within a Broader Inter-Kingdom Network

The virome is one component of a broader gut ecosystem that includes bacteria, fungi, archaea, the epithelial barrier, metabolites, and immune cells. Bacteria remain major metabolic and inflammatory effectors, while phages can change bacterial abundance, lysis, lysogeny, and accessory gene content. Fungi can alter bacterial communities, barrier integrity, and innate immune sensing, whereas archaea influence hydrogen transfer, methane production, redox balance, and syntrophic links with short-chain-fatty-acid-producing bacteria.

Integrated stool profiling in chronic hepatitis B-related liver fibrosis identified coordinated bacteriome, mycobiome, and virome changes, illustrating why a single-kingdom interpretation can miss disease-associated networks [26]. In colorectal cancer, viral–bacterial network models, prophage carriage within *Bacteroides fragilis*, and recent multi-kingdom profiling further support the idea that non-bacterial kingdoms may act through shared ecological networks rather than independently of bacterial hosts [6,27,28]. The causal strength of evidence remains unequal across kingdoms, and the available human data are still largely observational [26]. The inter-kingdom model should therefore guide hypothesis generation without implying that every detected organism is a driver.

### 2.3. Mechanism 1: Phage-Associated Ecological Remodeling in Carcinogenesis

**Figure 1:**
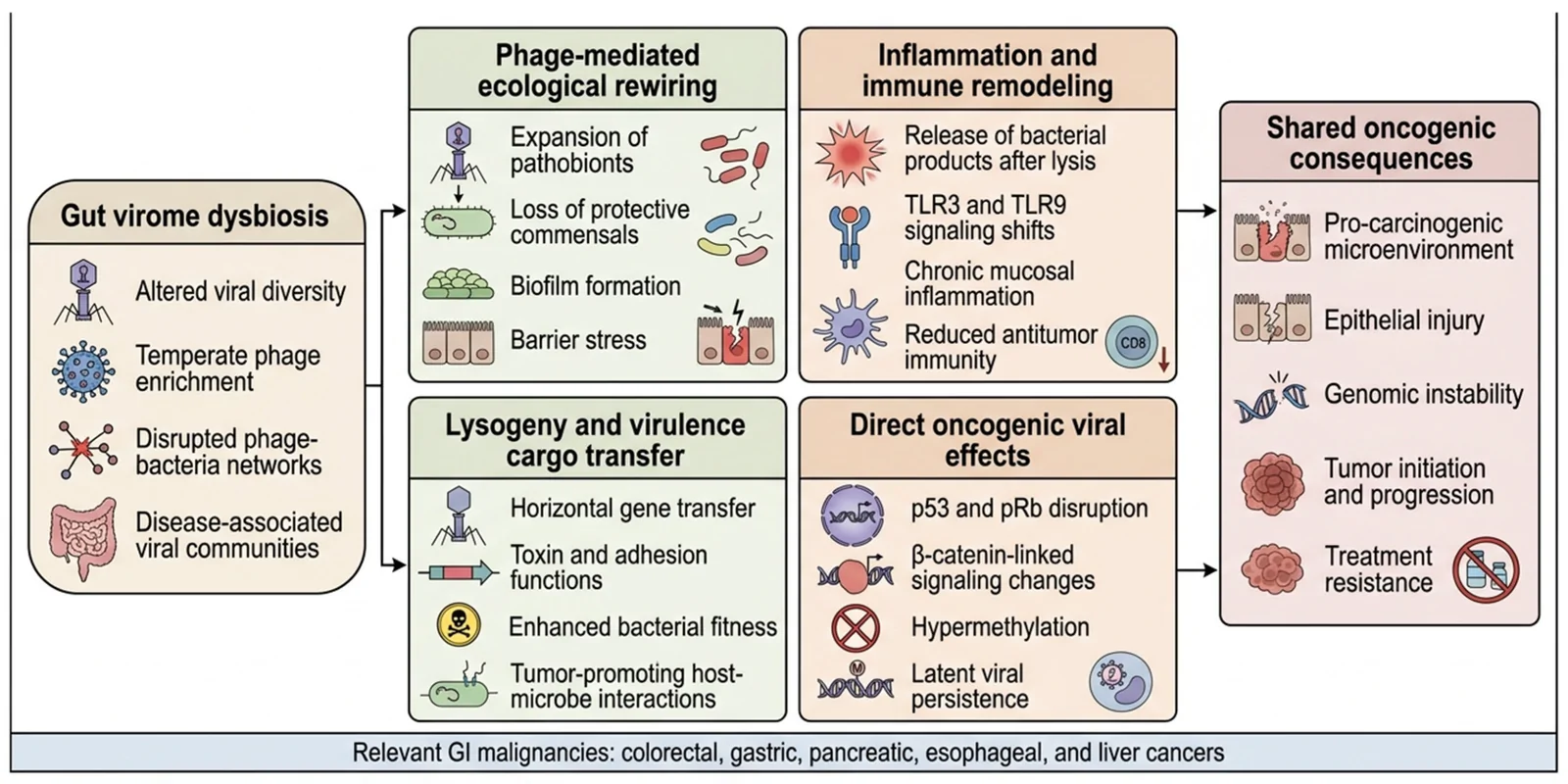
Proposed mechanistic routes linking gut virome dysbiosis to gastrointestinal carcinogenesis. This figure summarizes four connected routes by which the gut virome may contribute to gastrointestinal malignancies. Route 1 is phage-mediated ecological rewiring of bacterial communities, including expansion of pathobionts and loss of protective commensals. Route 2 is inflammation and immune remodeling, including altered Toll-like receptor signaling, chronic mucosal inflammation, and reduced antitumor immunity. Route 3 is lysogeny with transfer of virulence-associated functions, including horizontal gene transfer and enhanced bacterial fitness. Route 4 is direct oncogenic viral effects, including disruption of host cell-cycle control, tumor-suppressor and DNA-damage pathways, epigenetic reprogramming, viral integration or latency, and immune evasion.

The direction of causality should be stated carefully. Antibiotics, diet, inflammation, barrier injury, medication, and tumor-associated changes can reshape bacterial hosts and phage communities, while phage-mediated lysis, lysogeny, and gene transfer may in turn feed back on the dysbiotic ecosystem Figure 1. Current evidence therefore supports phages as potential modifiers of cancer-associated microbial states, not as established primary initiators of gastrointestinal cancer. Original stool metagenomic studies in colorectal cancer, adenoma, and pancreatic cancer, together with prophage–bacterium analyses, demonstrate association and biological plausibility but do not establish a human phage-initiated cancer pathway [5,6,9,20,29].

The strongest recurring theme is that bacteriophages may shape carcinogenic ecosystems indirectly by remodeling bacterial communities. In colorectal cancer, fecal viromes show disease-associated shifts in richness, composition, and phage–bacteria connectivity [5,8,20]. Viral and bacterial signals can move in opposite directions, with loss of health-linked bacteria and expansion of oral or inflammatory taxa such as *Fusobacterium nucleatum*, *Bacteroides fragilis*, *Porphyromonas* species, and other pathobionts [8,30].

Hannigan and colleagues argued that colorectal cancer-associated phages function as ecological hubs rather than direct human pathogens, opening niche space for driver bacteria that can form biofilms and sustain tumor-promoting inflammation [5]. Their study of 90 individuals found that the virome consisted mainly of temperate bacteriophages, while standard alpha- and beta-diversity measures did not clearly separate healthy, adenoma, and carcinoma groups. Instead, influential phage features emerged through machine-learning classification and network analysis. The viral model achieved a mean AUC of 0.792, close to the bacterial 16S model with an AUC of 0.809. The combined viral and 16S model did not significantly improve performance, showing that the virome is informative but not automatically additive [5].

Nakatsu and colleagues likewise found altered enteric viromes in colorectal cancer, increased bacteriophage richness, altered interactions between phages and oral bacterial commensals, stage-linked changes in viral profiles, and a survival-associated viral signature [8]. A 22-viral-genus classifier reached an AUC of 0.802 in the discovery cohort and 0.715–0.763 in three validation cohorts. A four-marker viral risk model was associated with reduced survival and remained prognostic after multivariable adjustment [8].

Zuo and colleagues extended this analysis across seven datasets from eight countries, including 462 colorectal cancer cases and 449 healthy controls. Phage-centered diversity was consistently higher in colorectal cancer, the correlation network between the virome and bacteriome was less intertwined, and genome-level viral classifiers showed within-study AUROCs ranging from approximately 0.65 to 0.92 and leave-one-dataset-out AUROCs of approximately 0.75–0.85 [20]. Performance was weaker in cohorts affected by long-term sample freezing or shallow sequencing, emphasizing the influence of technical history.

Recent work has extended the associative signal into precursor disease. A 2025 analysis of 419 adenoma cases and 552 controls identified 479 differential vOTUs, but performance in three external validation cohorts was modest, with AUCs of 0.61–0.65 [29]. A 2026 two-stage study identified two *B. fragilis* prophages, FU and ODE, that were enriched in CRC-associated isolates and validated their presence in 877 international metagenomes; however, the authors noted that the phages could be markers of increased *B. fragilis* abundance rather than causal drivers, unless lysogenic conversion is demonstrated [6].

This model helps explain why phage signals can track tumor biology even when many viruses are not themselves oncogenic. Phages regulate bacterial abundance, lysogeny, and gene flow. The relevant unit is often an interkingdom network in which viral shifts amplify bacterial virulence, mucosal injury, and inflammatory tone [1,2]. The available evidence supports this as a plausible route, but most human studies remain cross-sectional and cannot establish whether phage changes initiate bacterial dysbiosis or arise from a tumor-altered ecosystem.

### 2.4. Mechanism 2: Virome-Driven Inflammation and Immune Remodeling

The virome is not only a bacterial regulator; it is also an input to innate and adaptive immunity. Phage lysis releases bacterial products that act as inflammatory cues, while viral nucleic acids engage pattern-recognition receptors such as TLR3, TLR7, and TLR9 [1,2]. In this frame, the virome can amplify carcinogenesis by sustaining chronic inflammation, but it can also support antitumor immunity depending on context Figure 1.

The clearest functional evidence comes from Huang and colleagues, who studied antiviral depletion of the gut virome in a mouse colorectal cancer model [18]. An oral antiviral cocktail of ribavirin, lamivudine, and acyclovir reduced gut DNA and RNA viruses, increased tumor growth, shortened survival, and impaired the efficacy of 5-fluorouracil. Virome depletion reduced dendritic-cell and CD8-positive T-cell responses and downregulated the TLR3–IRF3–IFN-β signaling axis. Fecal microbiota transplantation restored virome features and improved 5-fluorouracil activity, while the TLR3 agonist poly(I:C) partly rescued immune activation and tumor control.

This study shifts the field from correlation toward functional evidence. The depletion, restoration, and TLR3-rescue experiments support a causal role for virome-dependent immune signaling in chemotherapy response within the mouse model [18]. However, the causal evidence is strongest in that experimental system and does not establish that spontaneous human tumorigenesis is initiated by virome disruption. The human component included only ten colorectal cancer patients, divided into responders and nonresponders, and should therefore be viewed as hypothesis-generating rather than as clinical validation.

Inflammation-linked virome effects also fit disease settings upstream of cancer Table 3. Reviews of the enteric virome have linked Caudovirales expansion, TLR9–MyD88 signaling, and IFN-γ-skewed immune responses to intestinal inflammation [1,2]. These observations connect virome changes in inflammatory bowel disease and colitis with later colorectal tumorigenesis, but prospective human studies are needed to determine whether virome alterations precede malignant transformation or reflect inflammation, medication, and altered barrier physiology.

### 2.5. Mechanism 3: Horizontal Gene Transfer, Lysogeny, and Virulence Cargo

Temperate phages can insert into bacterial genomes and may alter bacterial pathogenicity through lysogenic conversion, regulatory effects, or horizontal gene transfer. This mechanism is especially relevant when disease-associated phages are predicted to carry functions related to adhesion, toxins, lipopolysaccharide synthesis, or other processes that influence epithelial injury and inflammation Figure 1.

In Barrett’s esophagus and esophageal adenocarcinoma, rare phages were enriched for predicted exotoxin-related and lipopolysaccharide-biosynthesis functions, and temperate phages increased along the disease axis [21]. The most plausible interpretation is not that these phages directly transform epithelial cells, but that they may intensify inflammatory or invasive properties within the bacterial community. The small cohort and computational nature of the annotations require caution.

The same logic appears in pancreatic cancer. Zhang and colleagues identified 4863 vOTUs and 219 pancreatic cancer-associated vOTUs, including 65 enriched and 154 depleted in cases [10]. Cancer-enriched vOTUs were predicted to infect opportunistic or inflammatory bacterial families, including Veillonellaceae, Enterobacteriaceae, Fusobacteriaceae, and Streptococcaceae, whereas depleted vOTUs were more often associated with Ruminococcaceae, Lachnospiraceae, Oscillospiraceae, and other taxa that include butyrate-producing organisms. Enriched viral genomes carried predicted functions such as trimeric autotransporter adhesin and lysozyme, consistent with a possible role in bacterial adhesion, invasion, or community remodeling [10]. These functions remain hypotheses until verified experimentally.

The clearest experimental support for a pre-neoplastic route comes from the *Helicobacter pylori* study by Luo and colleagues. In tumor-prone mice, *H. pylori* infection triggered early expansion of temperate phages before overt colorectal cancer, with phage cargo enriched for genes such as flgJ, cwlJ, and sleB [19]. The authors proposed that inflammation and oxidative stress induce prophages, increasing horizontal gene transfer and bacterial lysis during a pre-neoplastic window. This offers a concrete route by which one microbe can rewire the broader gut ecosystem through the virome, although its relevance to human cancer remains to be established.

### 2.6. Mechanism 4: Direct Viral Carcinogenesis and Its Intersection with the Gut Virome

Any review of the gut virome in gastrointestinal cancer must keep direct viral oncology in view. Bacteriophages dominate stool metagenomes, but digestive cancers also include clear examples of eukaryotic viral carcinogenesis. These viruses matter not only as historical background but as proof that viral effects in gastrointestinal tissues can be causal, tissue-specific, and clinically actionable.

Epstein–Barr virus is the best-established example in gastric cancer. Foundational genomic and tissue studies showed that approximately 10% of gastric carcinomas are EBV-associated, with monoclonal viral genomes across tumor cells, restricted latency programs, and evidence of viral involvement before clonal tumor expansion [7,35]. EBV-positive tumors show EBER expression, latent viral transcripts, promoter hypermethylation of host tumor-suppressor genes, and pathway alterations involving PI3K–AKT and Wnt signaling [7,35].

Genomic analysis of gastric tissues from normal mucosa through low-grade dysplasia, high-grade dysplasia, and carcinoma further showed that EBV-associated gastric carcinomas and neighboring high-grade dysplasias share a common clonal origin and a single EBV strain within each patient [7]. EBV load rose sharply at the high-grade dysplasia and carcinoma stages, while genomic and epigenomic alterations converged on PI3K–AKT and Wnt signaling. Combined PI3K and Wnt inhibition reduced tumor growth in cell and xenograft models. The findings support a direct and clinically relevant role for EBV in a defined gastric cancer subtype, while also indicating that EBV may not initiate every early clone and may become widespread later in the precursor sequence [7].

In colorectal cancer, the picture is far less settled. JCV, HPV, CMV, and EBV have been investigated as possible cofactors, but the evidence is heterogeneous and often contested. For JCV in particular, meta-analytic evidence of T-antigen expression and paired mucosal studies illustrate the difficulty of separating persistent epithelial infection from incidental detection [36,37]. Detection of a viral sequence should not be equated with persistent infection, epithelial infection, or direct transformation.

In liver cancer, hepatitis viruses remain dominant etiologic agents. Newer virome-focused work suggests that broader viral exposure history may also carry biomarker value. Pan-viral serology identified distinct viral exposure patterns that predicted hepatocellular carcinoma and intrahepatic cholangiocarcinoma risk, and an HCC viral score correlated with liver injury markers and mortality [11]. This does not collapse HCC into a gut-virome disease, but it widens the frame from single-pathogen causality to host-wide viral ecology Figure 1.

## 3. Anelloviruses in Gastrointestinal Cancer

Anelloviruses are an increasingly recognized component of the human virome. The family Anelloviridae includes several genera, including Alphatorquevirus, Betatorquevirus, and Gammatorquevirus, commonly represented by torque teno virus, torque teno mini virus, and torque teno midi virus, respectively [38].

Unlike EBV, HBV, and HCV, anelloviruses have not been shown to directly transform human cells or cause cancer. Current evidence suggests that their viral load, diversity, and composition may reflect immune competence, chronic inflammation, tissue damage, and changes in host viral ecology associated with disease [39,40,41]. This distinction is essential because anelloviruses are widespread in healthy populations, with substantial species diversity [39]. Longitudinal analyses indicate that individuals carry highly personalized anelloviromes, which can remain relatively stable for years or decades [40]. TTV viremia is therefore common among healthy donors, although viral load varies substantially between individuals [41]. Detection alone has limited diagnostic significance.

By contrast, TTV viremia can have clinical monitoring value as an indirect marker of host immune control in transplant recipients and other immunologically compromised settings [41,42]. In this context, the measurement is not a cancer-specific test and should not be interpreted as evidence that anelloviruses cause malignancy.

**Figure 2:**
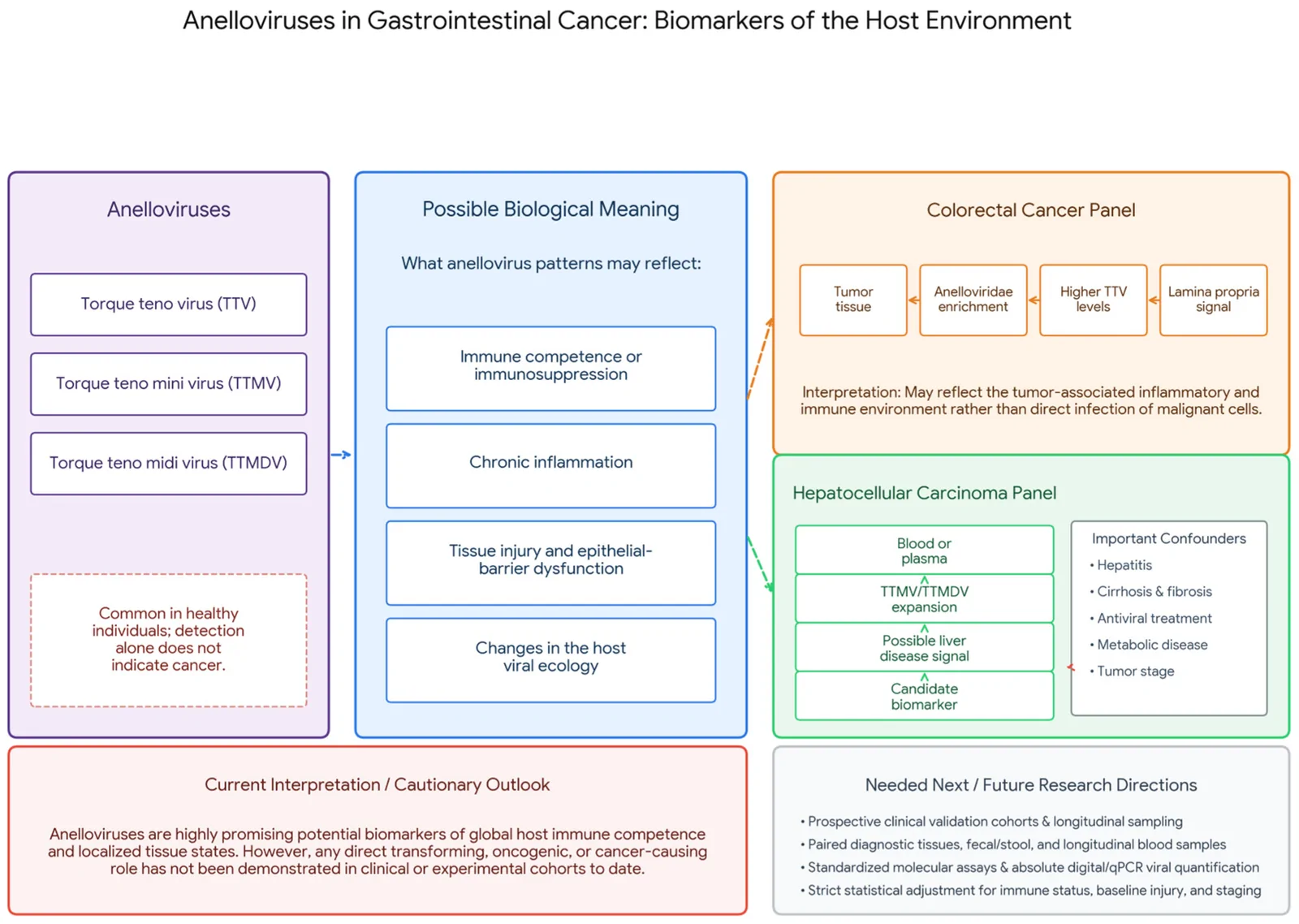
Anelloviruses in gastrointestinal cancer. Anelloviruses are widespread components of the human virome and are also detected in healthy individuals. Current evidence suggests that their abundance and composition may reflect immune status, inflammation, tissue injury, and altered viral ecology rather than direct oncogenic activity. In colorectal cancer, Anelloviridae enrichment and higher TTV levels have been reported in tumor-associated mucosa, particularly within the lamina propria [35]. In hepatocellular carcinoma and chronic liver disease, TTMV and TTMDV expansion may have biomarker value, although findings can be influenced by hepatitis, cirrhosis, treatment, and tumor stage [37,38,39,40].

In colorectal cancer, most virome evidence remains focused on phages and fecal samples [5,8]. Fecal profiles may not fully represent tumor-associated mucosal viral communities. A metagenomic analysis of virus-like particles from colorectal cancer tissue, adjacent mucosa, and healthy controls identified greater mucosal virome diversity and enrichment of the Anelloviridae family in colorectal cancer tissue Figure 2 [43]. Higher TTV levels were detected by quantitative PCR, while in situ hybridization showed a stronger signal predominantly in the lamina propria. Because the lamina propria is strongly influenced by tumor-associated inflammation and immune cells, the enrichment may reflect the local immune environment rather than a direct effect of malignant cells.

The most plausible interpretation is that anelloviruses are potential biomarkers and may modulate, or reflect, the tumor-associated mucosal immune environment. They may be linked to inflammation, epithelial-barrier dysfunction, and bacterial dysbiosis, but no direct role in promoting colorectal cancer has been demonstrated [38,42].

In HCC, anellovirus signatures may be influenced by chronic hepatitis, cirrhosis, fibrosis, systemic immune dysfunction, antiviral treatment, metabolic disease, and tumor stage. A quantitative study identified expansion of TTMV and/or TTMDV in the serum anellome of patients with HCV-associated HCC, although the cohort was small and cross-sectional [43]. Another study reported TTMV and/or TTMDV expansion in patients with severe liver disease, suggesting that blood-based anellome profiling may reflect systemic or liver-associated disease [44]. Together with detection of these viruses in a larger plasma cohort of HCC patients, these findings support investigation of TTV, TTMV, and TTMDV as additional biomarkers for risk stratification and longitudinal monitoring in chronic liver disease at risk for HCC [45]. Their specificity for cancer and clinical validity remain unproven.

Further work should use prospective cohorts, paired tissue, stool, and blood samples, absolute quantification, longitudinal sampling, and standardized molecular assays [41,45]. The primary question is whether anellovirus patterns add information beyond established measures of immune status, liver injury, viral hepatitis, and tumor burden.

## 4. Cancer-Specific Evidence

### 4.1. Standardized Levels of Evidence

The cancer-specific sections use the following evidence labels to distinguish study design from biological interpretation as described in Table 4.

A study may carry more than one label when it combines discovery, validation, and experimental components. Evidence level refers to the design and not automatically to the strength of a causal claim.

### 4.2. Cancer Types-Specific Evidence

#### 4.2.1. Colorectal Cancer

Level of evidence: Observational cohort and case-control evidence, mainly from retrospective stool metagenomics, with multicohort validation; selected mechanisms also have preclinical mouse support, but no human interventional study has established phage-driven carcinogenesis [5,6,8,18,20,32].

Colorectal cancer is the most developed setting for gut virome research. Across cohorts, the major findings are altered fecal virome composition, disease-associated phage diversity, disrupted interkingdom networks, and diagnostic or prognostic signals [5,8,20]. The direction of diversity is not fully consistent between studies, which likely reflects differences in sample preparation, sequencing strategy, reference databases, and cohort composition. The reproducible conclusion is therefore not a universal direction of diversity change, but a disease-associated shift in composition and network structure.

CRC studies support an interkingdom model. Altered phage signals occur alongside expansion of oral-derived or inflammatory bacteria such as *Fusobacterium nucleatum*, *Porphyromonas* species, and *Bacteroides fragilis*, together with depletion of protective or butyrate-producing organisms [30]. However, viral features do not always outperform bacterial features. In some cohorts, bacterial classifiers were stronger, while combined bacterial–viral panels offered only modest improvement [30]. Virome features should therefore be tested as complementary inputs rather than assumed to be superior biomarkers.

#### 4.2.2. Pancreatic Cancer

Level of evidence: Observational case-control and retrospective cohort evidence. Viral host assignments and functional annotations remain predominantly computational, and no study has established that a pancreatic cancer-associated vOTU causes disease [10]. A 2025 metagenomic study compared microbial signatures with CA19-9, but its single-center design and sample size require prospective validation [31].

The pancreatic cancer literature has a strong broader microbiome component and a newer direct virome component. Zhang and colleagues analyzed German and Spanish discovery cohorts and a Japanese validation cohort, identifying 4863 vOTUs and 219 disease-associated vOTUs [10]. The 20-vOTU model reached an average AUC of up to 0.879 in the discovery setting, while the Japanese validation cohort yielded an AUC of 0.734. Viral markers showed poor discrimination for several unrelated diseases, suggesting disease specificity, although the cohorts differed in age, prior treatment, antibiotic exposure, and geography.

The pancreatic cancer-enriched vOTUs were predicted to infect Veillonellaceae, Enterobacteriaceae, Fusobacteriaceae, and Streptococcaceae, including *Veillonella parvula*, *Fusobacterium nucleatum*, and *Escherichia coli*. Depleted vOTUs were more often associated with Ruminococcaceae, Lachnospiraceae, Clostridiaceae, Oscillospiraceae, and *Faecalibacterium prausnitzii*. The authors also reported predicted functions related to xenobiotic metabolism, trimeric autotransporter adhesin, and lysozyme [10]. These findings support a promising biomarker hypothesis, but host and function assignments require experimental validation.

#### 4.2.3. Gastric Cancer

Level of evidence: Observational tissue-based molecular evidence, including transcriptomic and metatranscriptomic studies with targeted laboratory validation. EBV-associated gastric carcinoma has substantially stronger causal evidence than broader gastric viral signatures [7,46,47].

Gastric cancer requires separation of direct tumor virology from gut-derived virome ecology. EBV-associated gastric carcinoma provides the strongest mechanistic evidence. EBV load rises at the transition to high-grade dysplasia and carcinoma, and high-grade dysplasia and carcinoma can share a monoclonal EBV strain within the same patient [7]. These findings are compatible with EBV infection preceding clonal expansion in a subset of tumors, but EBV does not explain gastric cancer as a whole.

The gastric virome also includes broader tissue signals, but those signals require careful interpretation. Viral reads in tumor transcriptomes or tissue metagenomes may reflect active infection, latent infection, integrated sequences, immune-cell carriage, or technical contamination. Direct evidence connecting these tissue signals to the fecal virome remains limited.

Recent tissue-resolved studies reinforce this distinction. Xiang and colleagues combined bulk and single-cell transcriptomic analyses of gastric tumors and adjacent tissue with RT-PCR and qRT-PCR validation of selected viral candidates [48]. Pereira and colleagues analyzed 238 gastric tissue samples and identified distinct metatranscriptomic virome clusters, including EBV-high tumors with a predominantly latent transcriptional program [46]. Zhou and colleagues additionally reported divergent blood and stool virome remodeling in gastric cancer, including marked blood Anelloviridae expansion and 67 provisional novel anellovirus species [47]. These studies strengthen the evidence for tumor- and circulation-associated viral signatures, but they remain observational and do not establish that the broader detected viruses are oncogenic.

#### 4.2.4. Barrett’s Esophagus and Esophageal Adenocarcinoma

Level of evidence: Observational case-control stool virome evidence from a very small cohort. The study included six controls, six Barrett’s esophagus patients, and only four patients with esophageal adenocarcinoma; predicted functions were not experimentally validated [21].

The direct gut virome evidence in Barrett’s esophagus and esophageal adenocarcinoma comes mainly from a small German study of six controls, six Barrett’s esophagus patients, and four patients with esophageal adenocarcinoma [21]. Caudovirales dominated all groups. Phage richness and Shannon diversity differed across disease states, and esophageal adenocarcinoma contained a higher proportion of temperate phages. Rare phages in disease samples were enriched for predicted functions related to exotoxins and lipopolysaccharide biosynthesis.

These observations are compatible with a model in which phage-mediated changes in bacterial function contribute to inflammation during the Barrett’s-to-adenocarcinoma sequence. They do not establish that the predicted genes are expressed, transferred to bacteria, or active in the esophageal mucosa. Larger longitudinal cohorts should compare stool, esophageal tissue, and host inflammatory markers before clinical claims are made.

#### 4.2.5. Hepatocellular Carcinoma and Intrahepatic Cholangiocarcinoma

Level of evidence: Mixed observational evidence, including stool virome profiling, serum serology, nested case-control sampling, and treatment-response cohorts, alongside established etiologic evidence for HBV and HCV. No human intervention study has shown that altering the resident gut virome prevents or treats HCC or iCCA [11,14,22,24,33,34].

HCC and iCCA occupy a distinctive position because the gut–liver axis is biologically important, while the most advanced viral biomarker studies measure systemic antiviral exposure rather than the gut-resident virome.

In stool, whole-genome shotgun metagenomics and viral-like-particle enrichment recovered overlapping but distinct viral fractions in HCC [14]. Of 1427 viral contigs at least 10 kb long, 1320 were shared, while 107 were specific to viral-like-particle enrichment and 48 to whole-genome shotgun sequencing. Both methods identified depletion of Faecalibacterium virus Taranis in HCC. Viral-like-particle enrichment also identified RNA viruses and additional Faecalibacterium phages, whereas whole-genome shotgun sequencing captured signals that may include free fragments or prophage-associated sequences. The study shows that method choice changes the apparent stool virome.

Pan-viral serology measures a different layer. Do and colleagues used phage immunoprecipitation sequencing to detect antibodies against more than 1280 viral strains in a large Thai cohort including HCC, iCCA, chronic liver disease, liver-fluke infection, and population controls [11]. The HCC score was based on 46 viral features and achieved training and testing AUCs of 0.954 and 0.778, respectively. The iCCA score was based on 51 features and achieved training and testing AUCs of 0.938 and 0.693. The HCC score was externally validated in a United States cohort with an AUC of approximately 0.76. Higher HCC scores were associated with liver injury markers and all-cause mortality in chronic liver disease.

Liu and colleagues used VirScan to identify a 61-virus exposure signature for early-onset HCC [49]. In a discovery cohort of 899 individuals and a longitudinal validation cohort of 173 at-risk patients, the signature achieved an AUC of 0.91 at baseline and 0.98 at diagnosis, with a median lead time of 8.8 years before clinical diagnosis. The signature included hepatitis viruses as well as EBV, CMV, and influenza A. These findings are clinically important but should be described accurately: serum antibody signatures measure host exposure history and immune memory, not the current gut virome. Recent work illustrates why prospective design and clinical comparators matter. In a nested case-control analysis within the prospective PLCO cohort, broad pan-serological signatures had poor discrimination for future liver cancer, with AUCs around 0.52–0.55; HCV was the main infectious exposure that remained associated with risk [33]. By contrast, a 2025 pan-microbial serology study in treated HCC patients identified a 23-peptide antigen score associated with treatment response and survival after adjustment for AFP, liver function, ALBI grade, and FIB-4, although the external validation effect was weaker than in the discovery cohort [34]. These results support serology as a possible adjunct to clinical risk stratification, not a replacement for established HCC surveillance and staging.

## 5. Biomarker and Clinical Applications

The most mature translational use of the gut virome is noninvasive biomarker development. Stool virome classifiers in colorectal cancer now have support from single-cohort studies, cross-cohort validation, and meta-analysis.

Nakatsu and colleagues reported a 22-viral-genus signature with a discovery AUC of 0.802 and validation AUCs from 0.715 to 0.763, together with a four-marker survival model [8]. Hannigan and colleagues found that a viral classifier performed similarly to a 16S bacterial model, with an AUC of 0.792 [5]. Chen and colleagues meta-analyzed 1282 fecal metagenomes across nine studies, identified 405 colorectal cancer-associated vOTUs, and reported leave-one-cohort-out AUCs of approximately 0.81–0.83, with independent validation as high as 0.906 [9].

The biomarker story is expanding beyond CRC. In pancreatic cancer, a random-forest model based on gut viral markers reached an AUC of 0.879 and retained signal in early-stage disease [10]. In HCC and iCCA, blood-based pan-viral serology yielded strong within-cohort discrimination and prognostic associations [11,50]. Barrett’s esophagus and esophageal adenocarcinoma studies remain too small for clinical claims, but they support the possibility that disease-linked virome states can be captured noninvasively [21].

Viral features should not be treated as mere add-ons to bacterial models. In some settings, they carry partly nonredundant information, while in others they add little beyond bacterial or clinical markers [5,30]. The best current use case is likely an integrated risk model combining virome, bacteriome, host markers, and clinical variables Table 5.

### Comparison with Current Clinical Standards

The clinical value of a virome biomarker depends on whether it adds information beyond the tests already used in practice. For CRC, large metagenomic studies generally use colonoscopy and histology as the reference standard, but direct head-to-head comparisons with CEA or FIT remain limited. The 2025 pooled analysis of 3741 stool metagenomes improved microbiome-only prediction across 18 cohorts, but it did not establish that a microbial or virome model should replace FIT or colonoscopy [32].

The clearest current comparison is in pancreatic cancer. In a 2025 case-control study, CA19-9 alone achieved an AUC of 0.825, whereas two fecal metagenomic models achieved AUCs of 0.923 and 0.853; combining microbial features with CA19-9 increased the AUCs to 0.977 and 0.953 [31]. These results are promising but require prospective, multicenter validation and comparison with contrast-enhanced CT, MRI, endoscopic ultrasound, and clinical staging. A microbiome model should be viewed as a potential adjunct to CA19-9 and imaging rather than as a standalone diagnostic test.

For HCC, serum viral or microbial signatures must be tested against AFP, liver function, ALBI grade, FIB-4, ultrasound, CT, and MRI. The HCC treatment-response study by Behrens and colleagues adjusted for several of these variables and found that the antigen score retained prognostic information, but the prospective PLCO analysis showed that broad prediagnostic pan-serology performed poorly [33,34].

## 6. Analytical Bottlenecks That Shape Interpretation

The technical bottleneck is multidimensional rather than limited to sequencing platform and depth. Pre-analytical factors include collection devices, time to freezing, storage temperature, freeze–thaw cycles, stool water content, tissue ischemia, blood contamination, and prior antibiotics, proton-pump inhibitors, chemotherapy, diet, or bowel preparation. These variables can alter both viral abundance and the bacterial hosts that structure the phageome [3,4].

Laboratory workflows introduce additional bias through host-cell depletion, virus-like-particle enrichment, filtration pore size, nuclease treatment, DNA versus RNA extraction, extraction chemistry, reverse transcription, whole-genome amplification, PCR-SISPA or multiple-displacement amplification, and library preparation. A 2026 methods comparison showed substantial differences in viral read recovery, bacterial contamination, contig completeness, and small circular ssDNA representation across these steps [16].

Bioinformatic choices then affect viral identification and quantification. Assembly may miss low-abundance genomes; read mapping depends on reference databases; viral prediction tools can disagree; host assignment may rely on CRISPR spacers, genome homology, or network inference; and functional annotation often remains incomplete. Long-read sequencing expands genome recovery, but it does not remove contamination, abundance, or classification problems [15]. Whole-genome shotgun sequencing and virus-like-particle enrichment also capture overlapping but non-identical fractions of the stool virome [14].

Finally, clinical confounding and statistical design shape apparent disease signals. Age, geography, diet, medication, bowel preparation, smoking, obesity, disease stage, tumor location, treatment exposure, sequencing batch, and case-control spectrum can all influence model performance. Viral dark matter and cohort-specific variation therefore remain central limitations [1,9]. These issues do not eliminate the field’s core signal, but they require harmonized protocols, technical controls, absolute or spike-in-based quantification where possible, paired specimens, and external validation before clinical deployment.

## 7. From Bench to Bedside

Therapeutic studies should be separated into distinct categories. Gut microbiome and virome modulation includes fecal microbiota transplantation, fecal filtrate or virome transfer, probiotics, prebiotics, defined bacterial consortia, and bacteriophage therapy; these approaches aim to alter a microbial ecosystem or its immune effects. Oncolytic virotherapy is different: engineered eukaryotic viruses are designed to infect tumor cells and activate antitumor immunity, not to remodel the resident gut virome. The two categories use different products, specimens, endpoints, and causal models and should not be presented as one translational pathway (Table 6).

Oncolytic virotherapy is the most clinically mature virus-based cancer strategy and has reached randomized phase III testing in a gastrointestinal cancer. Fecal microbiota transplantation and related whole-community interventions remain early-stage, while purified fecal filtrate or phage-directed approaches are exploratory. No human trial has yet isolated the gut virome as the active anticancer component. Microbiome-integrated oncology also includes distinct defined products—probiotics, prebiotics, live biotherapeutics, and bacterial consortia—whose composition, manufacturing, dosing, engraftment, and readouts differ from FMT [49]. VE800 and MET4 illustrate defined bacterial products rather than whole-community or virome transfer [51,52]. Cell-free fecal filtrate or fecal-virome transfer is a separate, phage-preserving strategy; current human evidence comes from non-cancer infection rather than GI oncology [51,53]. Related non-therapeutic studies reinforce this distinction: longitudinal work shows that the gut virome is individually structured and dynamic early in life, fecal virome signatures have diagnostic potential, and gut bacteriome–virome–metabolome networks can associate with immune status and hematological malignancy relapse [9,54,55,56]. These findings inform ecological and biomarker research but do not establish anticancer efficacy.

### 7.1. Oncolytic Viruses

Pexa-vec, or pexastimogene devacirepvec, advanced through encouraging early-phase work in liver cancer to the phase III PHOCUS trial. The study randomized 459 patients with advanced HCC to intratumoral pexa-vec followed by sorafenib or sorafenib alone [57]. The trial was stopped early for futility. Median overall survival was 12.7 months with the combination and 14.0 months with sorafenib alone. Response and disease-control rates were not improved, and serious adverse events were more frequent in the virus arm.

The most plausible explanation is pharmacological rather than conceptual: sorafenib arrests the cell cycle on which the thymidine-kinase-deleted virus depends for replication. The trial nevertheless provides a broader lesson. A biologically active virus does not automatically translate into clinical benefit, and combination design, sequencing, and immune context matter [57].

The pancreatic experience is more hopeful but earlier. LOAd703, an oncolytic adenovirus armed with CD40 ligand and 4-1BB ligand, was tested with gemcitabine and nab-paclitaxel in the single-center phase I and II LOKON001 trial [58]. Among 18 activity-evaluable patients, the objective response rate was 44%, the disease-control rate was 94%, and median overall survival was 9.3 months. Adenovirus-specific and effector-memory CD8-positive T-cell responses increased in most evaluable patients. The single-arm design, activity-evaluable population, and specialized intratumoral delivery limit causal interpretation, but the study supports further checkpoint-inhibitor combinations.

### 7.2. Microbiota and Virome Modulation

Microbiota-directed strategies are where the virome intersects most closely with mainstream immuno-oncology, even though the trials were not designed around the viral fraction. Two phase I studies showed that fecal microbiota transplantation from responder donors, given with anti-PD-1 therapy, could convert a subset of checkpoint-refractory melanoma patients into responders and reshape the tumor immune microenvironment [59]. These were not gastrointestinal cancer studies and did not attribute benefit to bacteriophages or other viruses. Viral screening in the trial was performed to exclude transmissible infectious agents, while mechanistic microbiome analyses focused mainly on bacteria.

The relevance to gastrointestinal oncology is therefore indirect. Transferred material includes a phage community, and the gut–immune axis is especially important in colorectal and hepatobiliary cancers. However, the available clinical data do not isolate the virome as the active component [59]. In ulcerative colitis, randomized FMT trials showed that recipient phageomes can shift toward donor profiles and that specific phage markers associate with remission, but the study did not test purified viral fractions [60]. A 2026 pilot of fecal filtrate transplantation in three hematological patients with fulminant Clostridioides difficile infection provides a safety and feasibility proof of concept for cell-free filtrates in immunocompromised patients, not evidence of anticancer efficacy [54].

Human GI oncology data now include the phase I NCT04130763 study, in which FMT capsules plus nivolumab produced a 20% objective response rate and 40% disease-control rate in ten anti-PD-(L)1-refractory patients; serial stool metagenomics and blood immune profiling supported donor-microbe engraftment and immune-response analyses [61]. RENMIN-215, a separate phase II single-arm study, combined FMT with tislelizumab and fruquintinib in 20 patients with refractory MSS metastatic colorectal cancer and reported a 20% objective response rate and 95% disease-control rate, but the contribution of FMT cannot be separated from the checkpoint inhibitor and antiangiogenic therapy [62]. NCT04729322 should likewise be described as a registered whole-community FMT and anti-PD-1 pilot for metastatic colorectal cancer, not as a virome-specific trial. These clinical results should not be used to infer phage efficacy unless viral components are measured and mechanistically linked to outcome.

The mechanistically detailed evidence comes from Huang and colleagues. In mice, antiviral treatment depleted gut viruses, expanded several phage groups, impaired dendritic-cell and CD8-positive T-cell responses, and reduced 5-FU efficacy. FMT restored aspects of the virome and antitumor response, while TLR3 agonism partly rescued the treatment effect [18]. These findings support virome-preserving strategies or TLR3 agonism as research directions, not established clinical therapies.

### 7.3. Bacteriophage Therapy

Bacteriophage therapy is the strategy most native to virome biology and, for now, the least clinically advanced in gastrointestinal oncology. A logical target is *Fusobacterium nucleatum* in CRC because of its association with tumor-promoting inflammation, immune evasion, and chemotherapy resistance. The FNU1 phage reduced *F. nucleatum*-induced growth, migration, autophagy, anti-apoptotic effects, and chemotherapy resistance in human colon and gastric cancer cell models [63].

The study did not include live-animal treatment experiments or human clinical data. It used mouse tissues and organoids to study receptor expression and human cancer cell lines for functional assays. Further testing in animal models is therefore required before clinical translation can be considered [63]. Registered phase I, II, or III trials of phage therapy in gastrointestinal malignancy are currently lacking in the evidence base considered here. Human phage-therapy trials completed to date have addressed infectious rather than oncologic indications. Major barriers include bacterial strain specificity, tumor access, phage neutralization, delivery, manufacturing, and the possibility that removing one bacterial host will not reverse a complex tumor-associated ecosystem.

**Table 6:** Translational studies by therapeutic category.

Therapeutic Category	Strategy and Setting	Evidence Level	Main Result	Main Limitation
Gut virome or microbiome modulation	Antiviral depletion, FMT, and 5-FU in a CRC mouse model, with a small human observational arm [18]	Preclinical intervention plus human correlation study	Antiviral depletion reduced gut DNA and RNA viruses, impaired dendritic-cell and CD8-positive T-cell responses and 5-FU efficacy; FMT and TLR3 agonism partly rescued the phenotype	Mouse model; no virome-specific clinical intervention; human arm *n* = 10
Gut virome or microbiome modulation	Donor FMT plus pembrolizumab in refractory melanoma [59]	Single-arm phase II; non-GI cancer	3 of 15 evaluable patients had objective responses, 3 had durable stable disease, and 6 had clinical benefit	Whole-community transfer; small non-randomized study; no virome-specific attribution
Gut virome or microbiome modulation	FMT-associated phageome shifts in two randomized ulcerative colitis trials [58]	Double-blind randomized FMT trials; non-cancer	Recipient phageomes shifted toward donor profiles and remission-associated vOTUs emerged across trials	Shotgun metagenomics without VLP purification; no purified viral fraction was tested
Gut virome or microbiome modulation	Cell-free fecal filtrate transplantation for fulminant CDI during chemotherapy-induced aplasia [54]	Prospective single-center pilot; *n* = 3; non-cancer	All three patients achieved clinical resolution by day +14 and the intervention was well tolerated	Single donor, no control group, no anticancer endpoint, and no clear general donor-phage effect
Phage-directed therapy	FNU1 targeting *Fusobacterium nucleatum* in colon and gastric cancer cell models and murine organoids [63]	Preclinical in vitro and ex vivo studies	Reduced *F. nucleatum*-driven growth, migration, autophagy, and chemotherapy resistance	No in vivo efficacy or human data; single bacterial strain
Direct oncolytic virotherapy	Pexa-vec plus sorafenib in advanced HCC [57]	Randomized phase III	Median overall survival was 12.7 versus 14.0 months; there was no survival benefit and the trial stopped for futility	Direct tumor infection, not resident gut-virome modulation; no tissue correlative data
Direct oncolytic virotherapy	LOAd703 plus gemcitabine and nab-paclitaxel in unresectable or metastatic pancreatic cancer [58]	Single-center phase I/II, single-arm; *n* = 21 safety, *n* = 18 activity	ORR was 44% and disease-control rate was 94% among activity-evaluable patients, with immune activation and responses in non-injected lesions	Small non-randomized activity-evaluable cohort; specialized delivery; chemotherapy contribution is unclear
Gut microbiome or virome modulation	FMT plus anti-PD-1 in refractory GI cancers: NCT04130763 and NCT04729322 [61]	Human phase I/II; whole-community intervention	NCT04130763 reported ORR 20% and DCR 40% in *n* = 10; NCT04729322 remains a registered CRC pilot without published efficacy results in the current evidence base	Small non-randomized studies; no virome-specific attribution

The clinical distinctions summarized in Table 7 should be preserved: microbiome-integrated interventions target the gut ecosystem, whereas oncolytic viruses target tumor infection and immune activation.

## 8. Discussion

The gut virome has emerged as a biologically relevant layer of gastrointestinal cancer research, but the evidence is not uniform across cancer types or biological measurements. The literature is best understood as three causal tiers. First, experimental studies provide functional support for specific mechanisms: antiviral depletion, fecal microbiota transplantation, and TLR3 agonism altered tumor growth and chemotherapy response in mice, while *H. pylori* infection induced early temperate-phage expansion before overt neoplasia [18,19]. Second, most human phage studies provide strong but still associative evidence, including disease-associated viral patterns, altered phage–bacteria networks, and stool-based classifiers in colorectal cancer [5,8,20]. These findings support the view that phages are ecological regulators rather than passive bystanders, but they do not prove that a particular phage initiates tumorigenesis.

The most coherent mechanistic synthesis is a sequence in which phages remodel bacterial communities, alter bacterial functions and gene flow, influence barrier injury and inflammation, and thereby change host immune tone. The experimental studies move this model beyond correlation, but their causal force remains strongest under defined conditions in mice. The *H. pylori* study emphasizes prophage induction and functional cargo during a pre-neoplastic inflammatory state, whereas the CRC antiviral-depletion model shows that virome disruption can impair immune-mediated chemotherapy efficacy [18,19]. Neither establishes that a human phage is a sufficient cause of cancer, and the predicted cargo in the *H. pylori* study still requires functional validation.

A separate tier comprises well-established viral carcinogenesis. EBV-associated gastric carcinoma demonstrates monoclonal viral involvement in dysplastic and malignant epithelial cells, rising viral load, and linked PI3K–AKT and Wnt pathway changes [7]. HBV and HCV are established etiologic agents of HCC, but their mechanisms are not interchangeable: HBV can contribute through recurrent integration and disruption of host genes, whereas HCV lacks genomic integration and acts through chronic inflammation, fibrosis, metabolic effects, and virus-specific signaling mechanisms [12,22,23,24]. These viruses should therefore not be grouped uncritically with stool bacteriophages or anellovirus signatures. They show instead that viral carcinogenesis may be direct, indirect, or mixed, depending on the virus, tissue, and stage of disease.

Anelloviruses occupy a different interpretive position. They are abundant and persistent in healthy people, so detection alone cannot establish cancer causation [39,40]. Their abundance and composition may nevertheless reflect immune control, inflammation, liver injury, and tissue-associated immune-cell states [41,43,64]. The most defensible near-term role for anelloviruses is therefore as biomarkers of host or disease state, particularly when measured longitudinally and interpreted with clinical context. Their interpretation should remain separate from both established oncogenic viruses and experimentally tested phage mechanisms.

The biomarker literature is promising but requires a stricter standard. High AUC values in discovery cohorts are not sufficient for clinical translation. Models must be compared against current clinical tests, bacterial microbiome models, host biomarkers, and disease-related confounders. They also require prospective validation across geography, age, diet, medication exposure, sequencing platforms, and disease stage. The lower testing performance of some serum scores relative to training performance illustrates the need for external validation [11].

AUC is a ranking metric and does not by itself show sensitivity, specificity, calibration, positive or negative predictive value, or clinical utility. AUCs from stool, tissue, plasma, and serum studies should not be compared as if they measure the same biological endpoint. Stage, tumor burden, disease prevalence, specimen matrix, case-control spectrum, and reference-standard choice can all change apparent performance. Future studies should therefore report sensitivity and specificity at clinically relevant thresholds, calibration, confidence intervals, decision-curve or net-benefit analyses, and stage-specific and externally validated results [9,10,31,46]. Biomarker performance should be treated as evidence of predictive utility, not as evidence of biological causation.

The trial landscape shows that mechanistic proximity to the resident virome and clinical maturity are different dimensions. Direct oncolytic viruses have reached late-phase testing because they are developed as conventional therapeutic products, but they are not resident gut-virome interventions. FMT, fecal filtrate, probiotics, prebiotics, and defined bacterial consortia alter whole microbial ecosystems and donot isolate phages as the active component. Bacteriophage therapy is the most direct virome-informed treatment concept but remains preclinical in gastrointestinal oncology. The near-term clinical value of the gut virome therefore lies less in virome-directed drugs than in virome-informed decisions: risk stratification, donor selection, treatment-response prediction, and identification of patients for mechanistic trials.

## 9. Future Directions

Future research should move gut-virome findings from association toward causal explanation through prospective longitudinal cohorts and mechanistic studies using isolates, organoids, immune co-cultures, and animal models; established oncogenic viruses should be treated as comparators rather than pooled with phage or anellovirus signals. Studies should harmonize stool and tissue collection, viral enrichment, extraction, sequencing, assembly, host assignment, and functional annotation, and report freeze–thaw history, host depletion, amplification, library preparation, spike-in controls, and read-mapping thresholds. Whole-genome shotgun sequencing and viral-like-particle enrichment should be considered complementary approaches, especially when RNA viruses, free particles, and prophage-associated sequences are all of interest [14,16]. Prospective studies should collect paired stool, mucosal, tumor, blood, and serum samples to determine whether stool signatures reflect tumor-adjacent mucosa, where tissue-associated anelloviruses reside, and whether antibody scores predict cancer independently of chronic disease. Candidate virome features should also be tested for added clinical value beyond bacterial metagenomics, inflammatory markers, circulating tumor markers, imaging, and established risk scores, with reporting that includes calibration, predictive values, confidence intervals, decision-curve analyses, and stage-specific performance, not AUC alone. Mechanistic work should validate predicted phage–bacterium interactions and virulence functions, including effects on *F. nucleatum*, *B. fragilis*, *E. coli*, and protective butyrate-producing bacteria. Anellovirus studies should use absolute quantification, strain-resolved sequencing, paired tissue and blood, and repeated pre- and post-treatment sampling to distinguish signals of immune suppression, liver injury, inflammation, or tumor burden, and to assess value beyond TTV load and established markers [41,45,65]. Early trials may be most informative when virome features guide interventions such as donor selection for FMT or fecal filtrate, preservation of the gut virome during chemotherapy, innate-sensing agonists, or phage cocktails targeting defined bacteria while measuring bacterial, fungal, archaeal, and viral communities and assessing safety, host range, neutralization, delivery, ecological effects, and manufacturing. Throughout, reports should distinguish resident gut viruses, tumor-associated viruses, circulating viral genomes, and serum antibody exposure rather than collapsing them into a single virome score.

## 10. Conclusions

The gut virome has moved from a neglected microbiome component to an emerging layer of gastrointestinal cancer biology. The evidence, however, falls into distinct causal tiers. Mouse intervention and rescue studies provide experimental support for selected virome–immune and virome–treatment pathways [18], while human phage studies in colorectal, pancreatic, and esophageal cancer mainly establish disease associations and biomarker potential [5,8,10,20,21]. These findings support phages as ecological regulators, but they do not yet demonstrate that a particular phage initiates human tumorigenesis.

Gastrointestinal malignancies are also shaped by established viral carcinogens. EBV-associated gastric carcinoma provides evidence of clonal, tissue-associated viral involvement and linked host-pathway changes [7]. HBV and HCV are established causes of HCC, although HBV-related integration and HCV-related inflammatory, metabolic, and virus-specific mechanisms differ [12,22,23,24,25,26]. These direct or mixed viral pathways should not be treated as mechanistically equivalent to associative stool phage signatures. Anelloviruses require greater caution: they are widespread in healthy populations and are not established oncogenic viruses, but their abundance and composition may provide information about immune competence, inflammation, tissue injury, or systemic disease [17,40,43].

**Figure 3:**
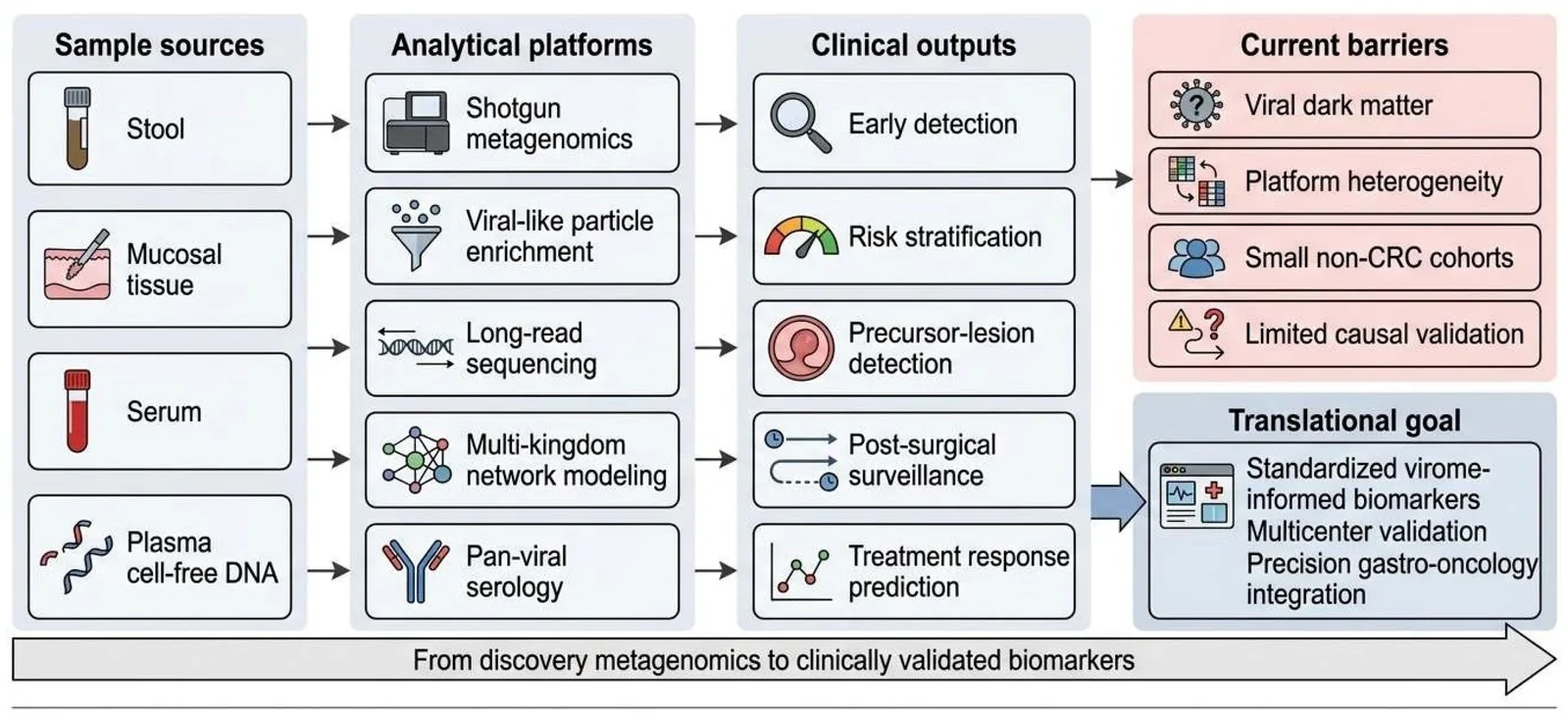
Clinical translation roadmap for virome informed gastrointestinal oncology. The figure outlines how viral measurements from stool, mucosal tissue, serum, and plasma can be processed through metagenomic, serologic, and multi-kingdom analytical platforms to support early detection, risk stratification, precursor-lesion detection, surveillance, and treatment response prediction. It also highlights the main barriers to implementation, including viral dark matter, method heterogeneity, limited mechanistic validation, and incomplete cross-cancer external validation.

The gut virome should be reconsidered as an emerging driver and translational layer of gastrointestinal cancer biology because it can reshape tumor-associated bacterial ecosystems, influence immune surveillance and treatment response, and generate measurable signals across stool, tissue, plasma, and serum. Its strongest human evidence currently supports biomarker and ecological roles, while experimental studies support selected causal mechanisms. Accordingly, virome-informed oncology should be viewed not as a replacement for established diagnostics or microbiome analysis, but as an additional framework for identifying high-risk states, precursor lesions, treatment-responsive patients, and potentially modifiable virus–bacterium–host interactions. The field’s priority is now to convert the roadmap shown in Figure 3 into prospective, standardized, mechanistically validated clinical studies.

Together, these lines of evidence support the gut virome as an emerging driver and biomarker layer in gastrointestinal cancers rather than a passive bystander, while also demanding careful separation of association, experimental mechanism, and established viral causation. The next task is to move from association-rich datasets to standardized, mechanistically anchored, clinically validated virome-informed oncology.

## Figures and Tables

**Table 1:** Partition of virome layer.

Virome Layer	Typical Specimen	What It Measures	Main Limitation
Luminal or stool virome	Stool, fecal virus-like particles, or fecal shotgun metagenomes	Shed virions, free viral nucleic acids, and phages associated with luminal bacteria	Sensitive to diet, transit time, medication, storage, and bacterial host composition; does not directly measure tumor tissue
Mucosa-associated virome	Endoscopic mucosa, adjacent non-tumor mucosa, or mucosal biopsies	Viruses associated with the epithelial surface, mucus, biofilms, and local immune cells	Sampling is spatially limited and vulnerable to blood, luminal carryover, and low-abundance contamination
Tumor-resident virome	Tumor tissue, tumor cells, or tumor microenvironment	Viral genomes or transcripts localized to malignant or infiltrating cells, together with tumor-associated phages	Requires careful controls for contamination, mapping errors, integrated sequences, and immune-cell carriage
Circulating or serologic virome	Plasma, serum, circulating viral nucleic acids, or antiviral antibodies	Systemic viral genomes or cumulative host exposure and immune memory	Does not represent the current resident gut virome; antibody signals may persist after infection has cleared

**Table 2:** Evidence tiers of viral carcinogenesis.

Evidence Tier	Representative Evidence	Appropriate Interpretation
Experimental support	In mice, antiviral depletion of gut viruses increased colorectal tumor growth and impaired 5-fluorouracil efficacy, whereas fecal microbiota transplantation and TLR3 agonism partly rescued these effects [18]. *Helicobacter pylori* infection also induced early expansion of temperate phages before overt neoplasia, although the proposed phage cargo remains to be validated [19].	Supports a mechanistic route under defined experimental conditions, but does not establish that a particular human phage causes cancer.
Strong association with unproven causation	Human studies report phage shifts, altered phage–bacteria networks, stage-linked signatures, and predicted host or functional changes in colorectal, pancreatic, and esophageal cancer [5,8,10,20,21].	Useful for biomarker development and hypothesis generation, but cross-sectional sampling, confounding, and computational annotations cannot distinguish cause from consequence.
Well-established viral carcinogenesis	EBV-associated gastric carcinoma shows monoclonal viral genomes in dysplastic and malignant epithelial cells, increased viral load, and linked PI3K–AKT and Wnt pathway alterations [7]. HBV and HCV are established etiologic agents of hepatocellular carcinoma [22]. HBV can contribute through recurrent integration and disruption of host genes [12,23], whereas HCV lacks genomic integration and acts through chronic inflammation, fibrosis, metabolic effects, and virus-specific signaling mechanisms [24,25].	Supports causal viral carcinogenesis in defined infections and tissues. The molecular route may be direct, indirect, or mixed, and should not be extrapolated to disease-associated stool phages or anellovirus detection.

**Table 3:** Integrated evidence map for gastrointestinal cancer virome research.

Cancer Setting or Route	Main Evidence	Translational Signal	Level of Evidence	Main Limitation
Colorectal cancer phage dysbiosis	Altered richness, composition, phage–bacteria networks, and CRC-associated prophages [5,6,8,20]	Stool diagnosis and exploratory prognosis	Observational cohorts and case-control studies; multicohort validation	No human causal intervention; assay and cohort heterogeneity
Adenoma–carcinoma sequence	Stage-linked viral changes and adenoma-associated vOTUs [8,29,30]	Potential progression markers	Observational case-control studies	External validation remains modest; temporal order is unresolved
Virome-driven immune remodeling	Antiviral depletion impaired tumor control and 5-fluorouracil efficacy; FMT and poly(I:C) partly rescued the phenotype [18].	Mechanistic basis for treatment-response studies	Preclinical intervention with a small observational human arm	Mouse model and small human component; no clinical intervention
Pancreatic cancer	Disease-associated vOTUs with computational host and function assignments [29].	Stool diagnostic models; possible complement to CA19-9 [31]	Observational case-control and retrospective cohorts	Host assignments and functions require experimental validation
Gastric cancer	EBV-associated clonal tissue changes and broader tissue viral signatures [7,32].	Molecular stratification and exploratory tissue biomarkers	Observational tissue studies; EBV mechanism supported by experimental models	Broader viral signatures are not equivalent to EBV causality
Barrett’s esophagus and esophageal adenocarcinoma	Temperate-phage enrichment and predicted functional changes [21].	Exploratory disease-state profiling	Observational case-control; *n* = 16 overall and *n* = 4 EAC	Very small cohort and unvalidated functional predictions
HCC and iCCA	Method-dependent stool virome changes and serum antibody signatures [11,14,33,34].	Risk stratification and treatment-response research	Mixed observational, nested case-control, and validation cohorts	Serum exposure measures are not resident gut virome; prospective findings are mixed
Phage lysis and viral nucleic-acid sensing	Bacterial-product release and TLR3, TLR7, and TLR9 pathways [1,2]	Candidate inflammatory and immune mechanisms	Mechanistically plausible; context-dependent	Cancer-specific causal links remain limited

**Table 4:** Evidence label of study design.

Evidence Label	Definition	What It Can Support
Preclinical	Cell-line, organoid, ex vivo, or animal experiments	Mechanistic plausibility, intervention effects, and causal testing under defined conditions
Observational cohort	Retrospective or prospective human samples collected without investigator-assigned treatment	Temporal associations, prognosis, biomarker performance, and external validation when appropriately designed
Observational case-control	Cross-sectional comparison of cases and controls, often using archived samples	Disease-associated signatures and hypothesis generation; limited temporal inference
Interventional	Non-randomized or randomized human treatment studies	Clinical efficacy or harm, subject to design, sample size, and comparator quality

**Table 5:** Biomarker landscape and level of evidence across gastrointestinal cancers.

Cancer	Specimen	Main Result	Level of Evidence	Clinical Comparator or Limitation
CRC	Stool metagenomics	Viral classifiers with multicohort and meta-analytic support [5,8,9,20]	Observational cohorts and case-control studies	Colonoscopy and histology were used as reference standards in large microbiome studies; direct comparison with CEA or FIT remains limited [32]
Pancreatic cancer	Stool metagenomics	219 disease-associated vOTUs; optimized AUC up to 0.879 [10]	Observational case-control and retrospective cohorts	Microbial signatures outperformed CA19-9 in one study, but combined models require prospective validation and were not compared head-to-head with imaging [31]
Gastric cancer	Tumor tissue and metatranscriptome	EBV-associated clonal changes and broader viral signatures [7,46,48]	Observational tissue studies; EBV-specific causal evidence is stronger	Endoscopy and histology remain clinical standards; noninvasive virome utility is unproven
Esophageal adenocarcinoma	Stool virome	Temperate-phage and predicted functional changes [21]	Observational case-control, very small cohort	Endoscopy and biopsy remain the diagnostic standard; no validated virome comparator
HCC and iCCA	Stool, plasma, and serum antibodies	HCC and iCCA viral scores and treatment-response signatures [11,34]	Mixed observational and validation cohorts	AFP for HCC, Ca 19.9 for iCCA, liver function, ALBI, FIB-4, ultrasound, CT, and MRI remain essential; prospective pan-serology performance was weak [33]
HCC	Serum antibody exposure	Early-onset HCC exposure signature with long lead time [49]	Retrospective discovery and longitudinal validation	Requires prospective comparison with surveillance algorithms and AFP-based care

**Table 7:** Human clinical trials in gastrointestinal cancer by therapeutic class.

Therapeutic Class	Trial or Population	Intervention	Clinical and Biological Readouts	Interpretation and Main Limitation
Gut microbiome modulation: FMT	NCT04130763; phase I; anti-PD-(L)1-refractory MSS GI cancers; *n* = 10, including eight gastric and two colorectal cancers [61]	Oral donor-FMT capsules followed by maintenance FMT plus nivolumab	ORR 20%; DCR 40%; serial stool metagenomics, blood immune and TCR profiling, and tumor PD-L1, MSI, and TMB assessment	Small single-center, single-arm study; whole-community transfer; no virome-specific attribution
Gut microbiome modulation: FMT	NCT04729322; pilot phase II; dMMR/MSI-H metastatic CRC after anti-PD-1 nonresponse	Colonoscopic responder-donor FMT followed by oral FMT capsules and re-introduction of pembrolizumab or nivolumab	ORR, safety, stool engraftment, and tumor or immune correlates	Registered pilot without published efficacy results in the current evidence base; whole-community intervention
Gut microbiome modulation: FMT plus ICI and antiangiogenic therapy	RENMIN-215; phase II; refractory MSS metastatic CRC; *n* = 20 [62]	FMT capsules plus tislelizumab and fruquintinib	ORR 20%; DCR 95%; median PFS 9.6 months and median OS 13.7 months; stool 16S profiling and peripheral-blood TCR sequencing	Small, single-center, single-arm study; the FMT contribution cannot be separated from the ICI and antiangiogenic components
Defined bacterial product or live microbial consortium	CONSORTIUM-IO, NCT04208958; phase I/II trial including gastric or gastroesophageal-junction cancer and MSS CRC cohorts [50]	VE800, a defined bacterial consortium, plus nivolumab after vancomycin conditioning	Safety, objective response, product engraftment, and ecological microbiome readouts	Defined product rather than FMT or virome transfer; GI-subgroup efficacy is not separately established in the available evidence
Virome-directed or phage-preserving therapy	No published GI-cancer efficacy trial; FNU1 remains preclinical and fecal-filtrate evidence is from non-cancer infection [54,63]	Phage targeting tumor-associated bacteria or cell-free fecal filtrate containing non-cellular microbial components	Preclinical tumor-cell effects and non-cancer feasibility; no GI-cancer response data	No human anticancer efficacy; host range, delivery, neutralization, manufacturing, and causal attribution remain unresolved [55]
Direct oncolytic virotherapy	PHOCUS; advanced HCC [57]	Intratumoral Pexa-Vec followed by sorafenib	Randomized phase III; median OS 12.7 versus 14.0 months; trial stopped for futility	Direct tumor infection and immune activation, not resident gut-virome modulation; no tissue correlative data
Direct oncolytic virotherapy	LOKON001; unresectable or metastatic pancreatic cancer [58]	Intratumoral LOAd703 plus gemcitabine and nab-paclitaxel	Phase I/II; ORR 44% among 18 activity-evaluable patients; immune activation and responses in non-injected lesions	Small single-arm study; chemotherapy contribution and activity-evaluable selection effects limit causal interpretation

## Data Availability

No new data were created or analyzed in this study. Data sharing is not applicable to this article.
